# Circulating CTRP1 in Adults with Type 2 Diabetes Mellitus: A Systematic Review and Meta-Analysis

**DOI:** 10.3390/ijms27156775

**Published:** 2026-07-29

**Authors:** Anirudh Suresh, Alexander Dallaway, Attia Mustafa, Lukasz Lagojda, Chris Kite, Ioannis Kyrou, Harpal S. Randeva

**Affiliations:** 1 Warwickshire Institute for the Study of Diabetes, Endocrinology and Metabolism (WISDEM), University Hospitals Coventry and Warwickshire NHS Trust, Coventry CV2 2DX, UK; 2 Warwick Medical School, University of Warwick, Coventry CV4 7AL, UK; 3 Faculty of Education, Health, and Wellbeing, School of Health and Wellbeing, University of Wolverhampton, Wolverhampton WV1 1LY, UK; 4 Institute for Cardiometabolic Medicine, University Hospitals Coventry and Warwickshire NHS Trust, Coventry CV4 7AL, UK; 5 Internal Medicine Department, Faculty of Medicine, Omar Al-Mukhtar University, Al-Bayda P.O. Box 919, Libya; 6 Sheffield Centre for Health and Related Research (SCHARR), School of Medicine and Population Health, University of Sheffield, Sheffield S1 4DA, UK; 7 Discoveries in Life Sciences Research Centre, Coventry University, Coventry CV1 5FB, UK; 8 Aston Medical School, College of Health and Life Sciences, Aston University, Birmingham B4 7ET, UK; 9 College of Health, Psychology and Social Care, University of Derby, Derby DE22 1GB, UK

**Keywords:** C1q tumour necrosis factor related proteins, CTRP, C1q tumour necrosis factor related protein 1, CTRP1, type 2 diabetes mellitus

## Abstract

Type 2 diabetes mellitus (T2DM) is typically associated with a dysregulated adipokine profile. C1q tumour necrosis factor-related protein 1 (CTRP1) is a relatively novel adipokine with protective metabolic effects, including improved insulin sensitivity. Clinical studies have increasingly highlighted a potential association between circulating CTRP1 and T2DM. Therefore, this systematic review and meta-analysis aimed to synthesise existing data on the association between circulating CTRP1 concentrations and T2DM in adults. MEDLINE, EMBASE, Cochrane Library, Web of Science, CINAHL and PsycInfo databases were searched for studies measuring circulating CTRP1 in adults with T2DM and controls. Eligible data were meta-analysed, reporting the mean difference (MD) and 95% confidence intervals (CIs) of circulating CTRP1 concentrations between T2DM cases and controls. The Revised Risk of Bias Assessment Tool for Nonrandomized Studies of Interventions (RoBANS 2) and the National Institutes of Health (NIH) quality assessment tool were utilised for study risk of bias and quality assessments, respectively. Eight studies (525 T2DM cases; 612 non-T2DM controls) were eligible for meta-analysis, showing significantly higher circulating CTRP1 concentrations in T2DM cases than controls (MD: 185.31 ng/mL; 95% CI: 140.02 to 230.60; *p* < 0.001). Most included studies were cross-sectional, and considerable heterogeneity was noted, with five studies having a high risk of bias in at least one RoBANS 2 domain. The MD remained relatively unchanged following a leave-one-out sensitivity analysis and exclusion of poor-quality studies. Meta-regression indicated that studies with a greater proportion of males (β = −188.68, *p* = 0.01) and higher mean body mass index (β = −23.96, *p* = 0.02) were associated with a lower MD. These findings indicate that circulating CTRP1 concentrations are significantly higher in adults with T2DM compared to non-T2DM controls. Future prospective and large-scale studies are required to delineate the nature of this association and explore the potential of CTRP1 as a biomarker for T2DM. PROSPERO registration ID: CRD420251137104.

## 1. Introduction

Type 2 diabetes mellitus (T2DM) is characterised by chronic hyperglycaemia and various degrees of insulin resistance [1,2]. Approximately 450 million people worldwide had T2DM in 2019, which represents an increase of almost 50% from 1990 [3]. Furthermore, T2DM is closely associated with obesity and metabolic syndrome and represents a well-established independent risk factor for cardiovascular disease [4,5], causing over one million deaths worldwide (the ninth leading cause of mortality) [6,7]. In addition, T2DM can lead to a spectrum of complications, such as nephropathy, neuropathy and retinopathy, with a significant impact on morbidity and quality of life [5]. Given the high morbidity and mortality associated with T2DM, significant work has focused on its underlying pathophysiological mechanisms and on identifying novel biomarkers that can support early diagnosis and/or targeted monitoring approaches in clinical practice.

The pathogenesis of T2DM involves a combination of insulin resistance and pancreatic beta-cell dysfunction, leading to relative insulin deficiency [1,8]. Obesity is closely linked to insulin resistance through complex hormonal and metabolic interactions, including dysregulated adipokine secretion [9]. Adipokines are secreted primarily by adipocytes and can enter the systemic circulation, reflecting the metabolic state of the different adipose tissue depots and exerting pleiotropic effects in various other target organs/tissues [1,10]. Adiponectin is a key adipokine with established anti-inflammatory, cardiovascular-protective, and insulin-sensitising effects, including increased glucose uptake and fatty acid oxidation, as well as reduced hepatic gluconeogenesis and lipolysis [9,11]. Several studies have shown that higher adiponectin levels are associated with a reduced T2DM risk [12,13]. Moreover, low levels of circulating irisin, which is secreted by skeletal muscles (myokine) and adipose tissue (adipokine), and appears to have insulin-sensitising effects, have been associated with T2DM [14]. Recently, adipokines that belong to a family of closely related adiponectin paralogues, known as the C1q tumour necrosis factor-related protein (CTRP) family, have been shown to have wide-ranging metabolic effects. Accordingly, research has increasingly focused on establishing their role in obesity-related cardio-metabolic diseases, such as T2DM [15,16,17].

CTRP1 is a 35kDa glycoprotein that belongs to the CTRP family of adiponectin paralogues and is primarily secreted by the stromal vascular fraction of white adipose tissue [18,19]. It structurally comprises an N-terminal signal peptide, a collagen-like domain, a short variable domain, and a C-terminal C1q-like globular domain [19,20]. Animal studies involving overexpression or knockout of CTRP1 have shown that it exerts protective metabolic effects, including improved insulin sensitivity and increased glucose and lipid utilisation [21,22,23,24,25]. Furthermore, mechanistic studies have shown that CTRP1 acts via AMP-activated protein kinase (AMPK) [24,26] and extracellular signal-regulated kinase 1/2 (ERK 1/2) [27] signalling pathways. Moreover, CTRP1 has been shown to increase insulin sensitivity through upregulation of the insulin receptor substrate 1 (IRS-1), a key receptor substrate in insulin signal transduction, by reducing phosphorylation of serine-1101 in IRS-1 [22]. This suggests that CTRP1 may have a compensatory role in reducing insulin resistance.

Considering this evidence, there is increasing research interest in exploring circulating CTRP1 as a potential biomarker in obesity, T2DM and cardiovascular diseases. As such, a growing number of clinical studies have reported a potential association between circulating CTRP1 and T2DM, but the existing evidence has not been systematically synthesised. Therefore, the present systematic review aimed to comprehensively synthesise the existing relevant studies and meta-analyse the available data on the difference in circulating CTRP1 concentrations between adults with T2DM and non-T2DM controls in order to explore its potential as an additional circulating biomarker in the context of T2DM.

## 2. Materials and Methods

The present systematic review and meta-analysis adheres to the Preferred Reporting Items for Systematic Reviews and Meta-analyses (PRISMA) guidelines [28] (the PRISMA checklist is presented in Supplementary Table S1) and was prospectively registered in the International Prospective Register of Systematic Reviews (PROSPERO Reference Number: CRD420251137104).

### 2.1. Search Strategy

Final literature searches were completed on 25 February 2026 in the MEDLINE, EMBASE, Cochrane Library, Web of Science, CINAHL and PsycInfo databases using a predefined search strategy developed by two reviewers (A.S. and L.L.). The Medline (via Ovid) search strategy is presented in Table 1, and this was adapted to the syntax and appropriate subject headings for each of the other databases, as presented in Supplementary Table S2. Citation chasing of included studies was also performed to ensure literature saturation.

### 2.2. Eligibility Criteria and Study Selection

Primary research studies published in English were eligible for inclusion if they measured circulating CTRP1 concentrations in adults (≥18 years) with T2DM and a control group without T2DM. There were no restrictions on publication date. Animal studies, case reports, case series, expert opinion articles, reviews and secondary analyses were excluded.

Following database searches, records were retrieved, and duplicates were removed. Study eligibility was assessed initially through title and abstract screening, which was followed by full-text review of all potentially eligible studies. Study selection was conducted independently by two reviewers (A.S. and L.L.) using the Covidence software (Covidence systematic review software, Veritas Health Innovation, Melbourne, Australia. Available at www.covidence.org, 2026) [29], with any conflicts resolved through consensus with a third reviewer (C.K.).

### 2.3. Data Extraction

Data extraction was conducted independently by two reviewers (A.S. and L.L. or A.M.) using the Covidence software [29], with validation by a third reviewer (C.K.). A pre-specified, standardised data extraction proforma was used to extract data from eligible studies. Extracted data included year of publication, country of study origin, study design/setting, pertinent baseline characteristics [e.g., age, sex distribution, and body mass index (BMI)], T2DM diagnostic criteria, sample sizes for the T2DM and control groups, circulating CTRP1 concentrations, and CTRP1 measurement method. Attempts to contact the corresponding study author(s) were made if pertinent data were missing and/or reported as median and interquartile range (IQR). Where responses to these requests were not received, median and IQR values were converted to the corresponding mean and standard deviation (SD) values using previously outlined formulae [30,31]. Where responses to the requests for CTRP1 data were not received, but these were available in figure format within the published article, CTRP1 values were extracted from the available figures using a plot digitiser [32,33].

### 2.4. Quality Assessment

Risk of bias assessment was conducted independently by two reviewers (A.S. and C.K.) for all included studies using the Revised Risk of Bias Assessment Tool for Nonrandomized Studies of Interventions (RoBANS 2) [34]. This validated tool includes the following eight domains: comparability of the target group; target group selection; confounders; measurement of exposure; blinding of assessors; outcome assessment; incomplete outcome data; and selective outcome reporting. Quality assessment was also performed using the National Institutes of Health (NIH) quality assessment tool [35] for the appropriate study design. This quality assessment was performed since study quality and risk of bias are complementary and not interchangeable, and thus, evaluating both was necessary to fully appraise methodological safeguards and the likelihood that bias influenced study findings [36]. Any conflicts in these risk of bias and quality assessments were resolved through consensus and discussion with a third reviewer (A.M.). Risk of bias plots were generated using the online robvis tool [37]. Certainty of evidence was formally assessed using the Grading of Recommendations Assessment, Development, and Evaluation (GRADE) approach [38,39].

### 2.5. Statistical and Meta-Analysis

Meta-analytical modelling, statistical analyses and visualisations were performed using the meta package [40,41,42] in the RStudio environment (R version 4.5.1, RStudio 2025.05.1+513 “Mariposa Orchid” Release for Windows). Results are reported as the mean difference (MD) in circulating CTRP1 concentrations in ng/mL units between T2DM cases and non-T2DM controls and the corresponding 95% confidence intervals (CI).

A random-effects model, using the inverse variance method for pooling effect sizes, was used for the meta-analysis. We used the raw MD since CTRP1 concentrations were measured by the same assay and were reported in the same units across studies (ng/mL), allowing the pooled estimate to retain its original biological scale and aiding interpretation. The restricted maximum-likelihood (REML) estimator was used to estimate between-study heterogeneity, and the Hartung–Knapp adjustment was applied to provide a more conservative CI for the summary effect size. A sensitivity analysis was performed in which studies with a “poor” quality assessment based on the NIH quality assessment tool were removed. To identify outliers, a Baujat plot was used to identify studies that greatly influenced the pooled result and heterogeneity. Additionally, a series of influence analysis diagnostics [43] were performed using the dmetar package [42,44]. A leave-one-out sensitivity analysis was also performed.

Study heterogeneity was assessed using Cochran’s Q and *I*^2^ statistics. For the Q-test, *p* < 0.1 was considered significant. For the *I*^2^ test, heterogeneity was considered as either moderate (*I*^2^: 30–60%), substantial (*I*^2^: 50–90%) or considerable (*I*^2^: 75–100%) based on established thresholds [45]. Heterogeneity was investigated using subgroup analyses based on T2DM diagnostic criteria and country of study origin. Additionally, univariable meta-regressions were conducted to examine whether mean age, proportion of male participants, and mean BMI (calculated as pooled means across T2DM and control groups within each study) were associated with variation in effect estimates.

Publication bias was explored using Egger’s regression [46] and visual inspection of a funnel plot. Where there was evidence of publication bias, a Duval and Tweedie’s trim-and-fill procedure [47] was conducted to produce an adjusted pooled effect estimate by imputing additional studies to account for publication bias. A sunset plot was produced to investigate the power of each included study to detect the meta-analytic effect size [48]. A *p*-curve analysis was performed to test for evidential value, detect potential data manipulation and publication bias [49].

## 3. Results

### 3.1. Eligible Study Selection and Characteristics

**Figure 1:**
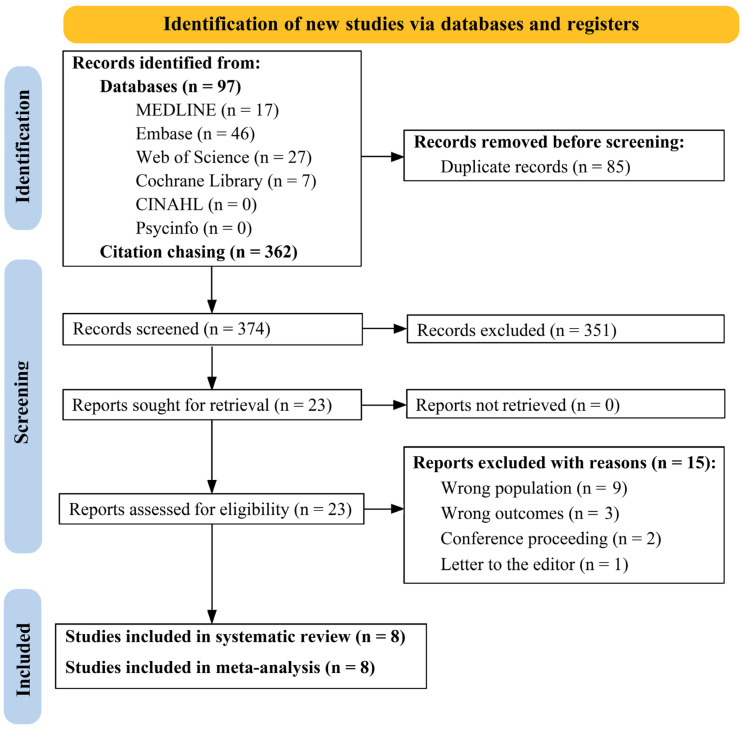
PRISMA flow diagram outlining study selection for the present systematic review and meta-analysis.

Figure 1 presents the PRISMA flow diagram for the studies identified for this systematic review. In brief, 459 records were identified from the performed database searches and citation chasing. These were imported to Covidence, where 85 duplicate records were removed, leaving 374 studies for title and abstract screening. Following title and abstract screening, 351 studies were excluded as they did not meet the predefined eligibility criteria, resulting in 23 studies for full-text review. During full-text review, 15 studies were excluded with reasons (Figure 1), resulting in a total of eight studies eligible for inclusion. The key characteristics of these eight included studies are outlined in Table 2.

### 3.2. Risk of Bias and Quality Assessment

**Figure 2:**
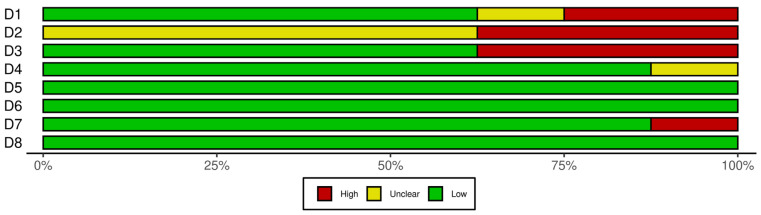
Summary plot for the risk of bias assessment of all eight eligible studies included in the present systematic review and meta-analysis, assessed using the Revised Risk of Bias Assessment Tool for Nonrandomized Studies of Interventions (RoBANS 2) [34]. D1: comparability of target group; D2: target group selection; D3: confounders; D4: measurement of intervention/exposure; D5: blinding of assessors; D6: outcome assessment; D7: incomplete outcome data; D8: selective outcome reporting.

**Figure 3:**
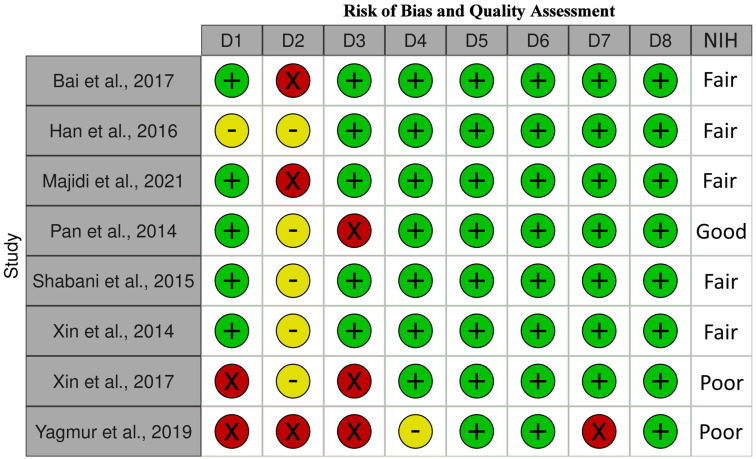
Traffic light plot for the risk of bias assessment for each of the eight eligible studies included in the present systematic review and meta-analysis [22,50,51,52,53,54,55,56], assessed using the Revised Risk of Bias Assessment Tool for Nonrandomized Studies of Interventions (RoBANS 2) [34] and overall ratings using the National Institutes of Health (NIH) quality assessment tool [35]. D1: comparability of target group; D2: target group selection; D3: confounders; D4: measurement of intervention/exposure; D5: blinding of assessors; D6: outcome assessment; D7: incomplete outcome data; D8: selective outcome reporting; NIH: National Institutes of Health quality assessment tool overall ratings. For each study, “+” in a green circle indicates low risk of bias; “-” in a yellow circle indicates unclear risk of bias; “X” in a red circle indicates high risk of bias in each respective domain.

The summary of the risk of bias assessments based on the ROBANS 2 is presented in Figure 2. In brief, there were five studies (62.5%) that had a low risk of bias from target group comparability, while two studies [22,56] (25%) had a high risk of bias, due to the inclusion of broad, non-specific illnesses that could vary between groups, and one study [51] (12.5%) had an unclear risk of bias for this domain. For participant selection, three studies [50,52,56] (37.5%) had a high risk of bias, as participants were consecutively recruited without ensuring the representativeness of the population, whilst the risk of bias for the rest of the studies [22,51,53,54,55] (62.5%) was unclear. Appropriate confounder adjustment was done in five studies (62.5%), giving these a low risk of bias, whilst the remaining three studies [22,53,56] (37.5%) had a high risk of bias. For the measurement of the exposure domain, the risk of bias was unclear in one study [56] (12.5%), which failed to specify the applied diagnostic criteria for T2DM, whereas these were clearly reported in all other studies (87.5%), giving them a low risk of bias in this domain. All studies had a low risk of bias regarding the blinding of assessors, outcome assessment, and selective outcome reporting domains. Although none of the included studies were blinded, this was not deemed to significantly affect the study outcome regarding the circulating CTRP1 measurements, which were performed using a validated CTRP1 enzyme-linked immunosorbent assay (ELISA). Finally, regarding the incomplete data reporting domain, one study [56] (12.5%) was deemed to have a high risk of bias due to incomplete data for the control group, while the rest had a low risk of bias. Overall, one study was rated as “good” [53], five as “fair” [50,51,52,54,55] and two as “poor” [22,56] according to the NIH quality assessment tool. For all included studies, the detailed risk of bias assessment for each ROBANS 2 domain and the overall ratings using the NIH quality assessment tool are presented in Figure 3. Collectively, the identified risks of bias and study quality may have influenced the final pooled estimates primarily through differences in participant selection, target group comparability, confounder adjustment, and missing data. Although the overall findings suggest higher circulating CTRP1 concentrations in T2DM, the pooled estimate should be interpreted with caution given the potential contribution of these methodological limitations to between-study heterogeneity. This has been reflected in the downgrading of the certainty of evidence using GRADE (Table 3).

### 3.3. Circulating CTRP1 Concentrations in T2DM Cases Versus Controls

All eight eligible studies identified for the present systematic review were meta-analysed, including a total of 525 adults with T2DM and 612 non-T2DM controls. To be able to meta-analyse the CTRP1 data from all eligible published papers, for three studies [52,54,56], a plot digitiser was used to extract the pertinent CTRP1 data from relevant published figures. For one study [56], the reported median BMI and age could not be converted to mean values using established conversion formulae [30,31] due to missing lower and upper quartile values. Therefore, the median values were used as an estimate for the mean, as previously described for sample sizes greater than 25 [57]. The study by Shabani et al. [54], in addition to a T2DM and a healthy control group, had a non-T2DM group with non-alcoholic fatty liver disease (NAFLD) and a group with both T2DM and NAFLD (T2DM-plus-NAFLD group). Taking into account that there is consistent evidence that at least half of all adults with T2DM have NAFLD [58], for the present meta-analysis, the T2DM and T2DM-plus-NAFLD groups were aggregated and compared with the healthy control group.

**Figure 4:**
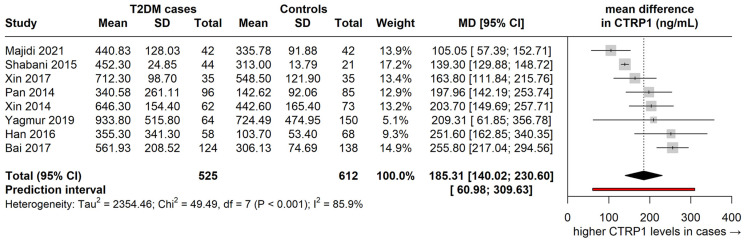
Forest plot presenting the meta-analysis for the mean difference in circulating CTRP1 concentrations in adults with type 2 diabetes mellitus (T2DM) compared to non-T2DM controls from the eight eligible studies [22,50,51,52,53,54,55,56]. CI: confidence interval; MD: mean difference; SD: standard deviation.

**Figure 5:**
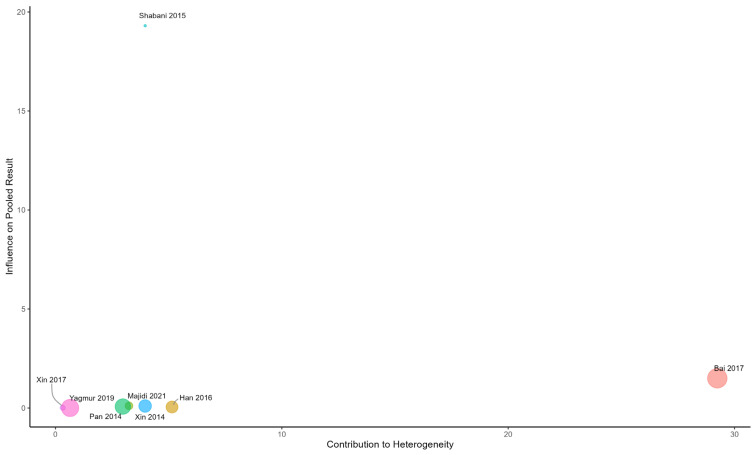
Baujat plot analysis of all eight eligible studies included in the present systematic review and meta-analysis [22,50,51,52,53,54,55,56]. Each circle represents an individual study, with the circle size representing the weight of the corresponding study.

**Figure 6:**
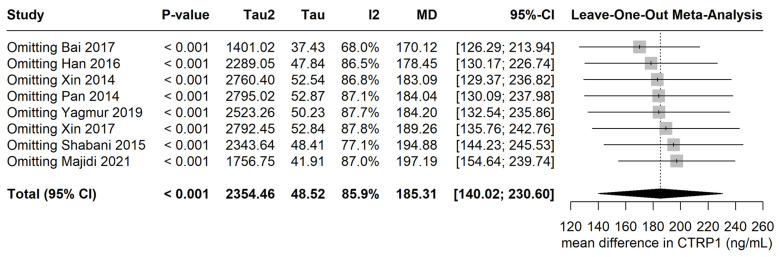
Forest plot showing the leave-one-out sensitivity analysis for the eight eligible studies included in the present meta-analysis [22,50,51,52,53,54,55,56]. CI: confidence interval; MD: mean difference.

According to the performed meta-analysis, the circulating CTRP1 concentrations were significantly higher in the T2DM cases (n = 525) compared to those in non-T2DM controls (n = 612) with a MD of 185.31 ng/mL (95% CI: 140.02 to 230.60; *p* < 0.001; Figure 4). All included studies had higher CTRP1 concentrations in adults with T2DM compared to those in their respective control groups. Considerable heterogeneity was identified between the eight eligible studies (*I*^2^ = 85.9%; τ^2^ = 2354.46). Visual inspection of the Baujat plot revealed that the Bai et al. study [50] contributed greatly to heterogeneity and was positioned away from the other studies (Figure 5). The influence analysis confirmed the Bai et al. study [50] as an outlier. Once removed as part of a sensitivity analysis, there was a negligible change to the pooled effect size (MD: 170.12 ng/mL; 95% CI: 126.29 to 213.94); however, there was a noticeable reduction in heterogeneity (*I*^2^ = 68.0%; τ^2^ = 1401.02) (Figure 6). Removing the two studies [22,56] with a “poor” quality assessment caused a negligible increase in the pooled effect size (MD: 188.16 ng/mL; 95% CI: 124.83 to 251.49) and in heterogeneity (*I*^2^ = 89.7%; τ^2^ = 3058.67). An additional sensitivity analysis using only the T2DM-only group from Shabani et al. [54] produced a similar pooled estimate (MD: 183.81 ng/mL; 95% CI: 136.97, 230.66; *I*^2^ = 87.8%) to the pooled estimate of the primary analysis, indicating that the latter was not dependent on the inclusion of both the T2DM and the T2DM-plus-NAFLD groups of this study.

### 3.4. Subgroup Analysis

Subgroup analyses were performed based on the applied T2DM diagnostic criteria in each of the eight included studies and based on their country of origin. No subgroup analysis was performed based on the applied CTRP1 measurement method since all studies used the same CTRP1 ELISA assay (Table 1).

**Figure 7:**
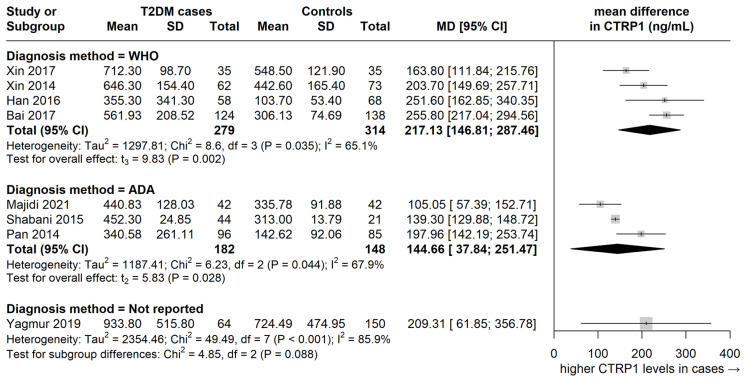
Forest plot presenting the subgroup analysis by the type 2 diabetes mellitus (T2DM) diagnosis criteria applied in each of the eight studies included in the present meta-analysis [22,50,51,52,53,54,55,56]. ADA: American Diabetes Association; CI: confidence interval; MD: mean difference; SD: standard deviation; WHO: World Health Organization.

For subgroup analysis based on the applied T2DM diagnostic criteria, there was no significant difference (*p* = 0.088) in the pooled effect size between the four studies [22,50,51,55] using the World Health Organization (WHO) diagnostic criteria for T2DM (MD: 217.13 ng/mL; 95% CI: 146.81 to 287.46), the three studies [52,53,54] using the American Diabetes Association (ADA) criteria (MD: 144.66 ng/mL; 95% CI: 37.84 to 251.47) and the one study [56] that did not report the applied T2DM diagnostic criteria (MD: 209.31 ng/mL; 95% CI: 61.85 to 356.78) (Figure 7). Heterogeneity was substantial in both the WHO (*I*^2^ = 65.1% [τ^2^ = 1297.81]) and ADA (*I*^2^ = 67.9% [τ^2^ = 1187.41]) subgroups. Heterogeneity was not present in the “not reported” subgroup, as only one study [56] was included.

**Figure 8:**
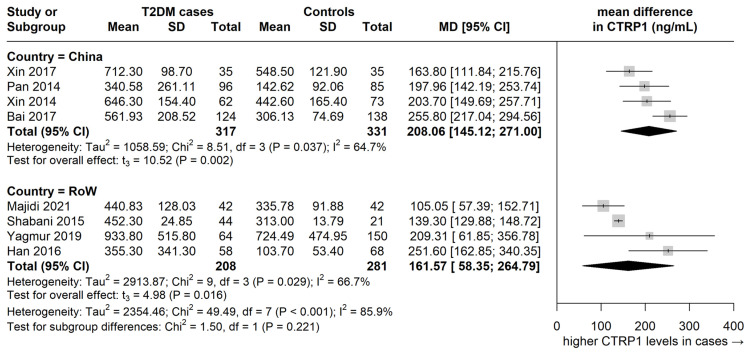
Forest plot presenting the subgroup analysis by the country of origin of the eight studies included in the present meta-analysis [22,50,51,52,53,54,55,56]. The four studies from China were grouped together, while there were also four studies, namely two from Iran, one from Republic of Korea (South Korea) and one from Germany, which were grouped as the rest-of-the-world subgroup. CI: confidence interval; MD: mean difference; RoW: rest of the world; SD: standard deviation.

For subgroup analysis based on the country of origin of each study, the four studies from China [22,50,53,55] were grouped together, while there were also four studies, namely two from Iran [52,54], one from the Republic of Korea (South Korea) [51] and one from Germany [56], which were grouped as the rest-of-the-world subgroup (Figure 8). No significant difference (*p* = 0.221) was noted in the effect size between the four studies that were conducted in China (MD: 208.06 ng/mL; 95% CI: 145.12 to 271.00) and the four studies from the rest of the world (MD: 161.57 ng/mL; 95% CI: 58.35 to 264.79) (Figure 8). Heterogeneity was substantial in both the China (*I*^2^ = 64.7% [τ^2^ = 1058.59]) and the rest-of-the-world (*I*^2^ = 66.7% [τ^2^ = 2913.87]) subgroups. Supplementary Figure S1 presents the global distribution of the eight included studies based on their country of origin.

### 3.5. Meta-Regression Analysis

**Figure 9:**
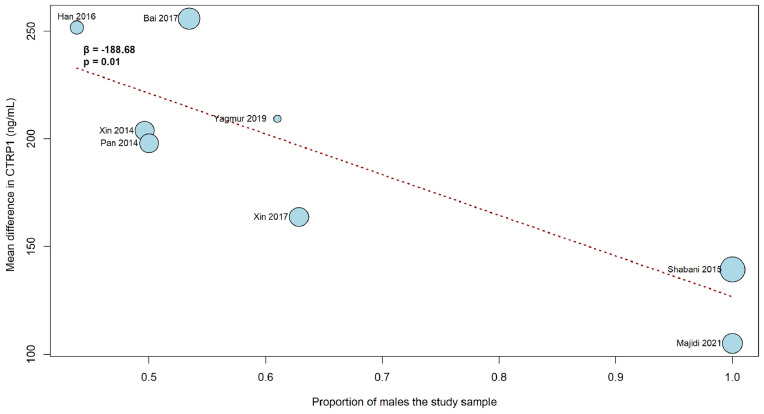
Bubble plot showing the relationship between proportion of males in study samples and mean difference in CTRP1 concentrations between T2DM and control groups. Each bubble represents an individual study [22,50,51,52,53,54,55,56], with bubble size representing study weight. The dashed line represents the fitted meta-regression. A higher proportion of males was associated with smaller between-group differences in CTRP1 concentrations (β = −188.68, *p* = 0.01).

**Figure 10:**
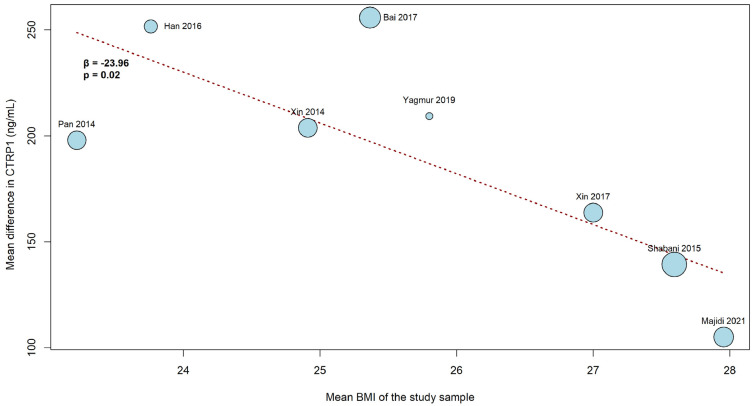
Bubble plot showing the relationship between mean body mass index (BMI) of study samples and mean difference in CTRP1 concentrations between T2DM and control groups. Each bubble represents an individual study [22,50,51,52,53,54,55,56], with bubble size representing study weight. The dashed line represents the fitted meta-regression. Higher mean BMI was associated with smaller between-group differences in CTRP1 concentrations (β = −23.96, *p* = 0.02).

Studies with a greater proportion of males (β = −188.68, *p* = 0.01; Figure 9) and higher mean BMI (β = −23.96, *p* = 0.02; Figure 10) were associated with a lower MD in circulating CTRP1 concentrations in T2DM cases versus controls. The effect of sample mean age was not significant (β = −2.69, *p* = 0.53).

### 3.6. Publication Bias

**Figure 11:**
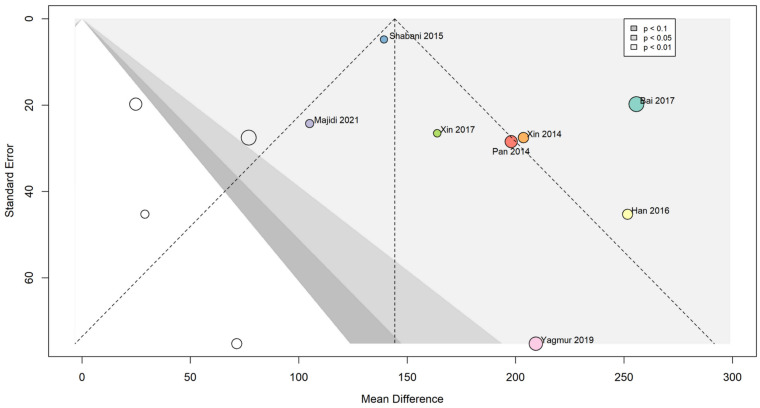
Funnel plot of all studies included in the present meta-analysis (coloured circles) [22,50,51,52,53,54,55,56] and imputed studies by Duval and Tweedie’s Trim and Fill procedure (white circles).

Egger’s regression test indicated a low risk of publication bias (t = 1.94, *p* = 0.10). However, visual inspection of the funnel plot indicated that asymmetry was present (Figure 11). Duval and Tweedie’s Trim and Fill procedure was therefore conducted, which added four imputed studies to the left. The adjusted MD was reduced to 144.26 ng/mL (95% CI: 92.55 to 195.96; *p* < 0.001).

**Figure 12:**
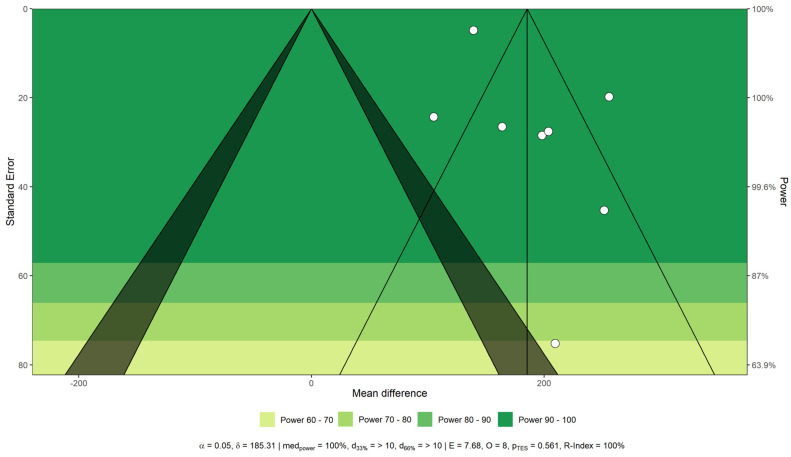
Sunset plot of all studies included in the present meta-analysis. Each white circle represents an individual study.

The power-enhanced sunset plot (Figure 12) showed that the included studies were well powered to detect the observed effect, with seven studies exceeding 90% power. The median power was 100%. The expected number of statistically significant studies (7.68) was consistent with the observed number (8.00), suggesting no evidence of excess significance (*p* = 0.561).

**Figure 13:**
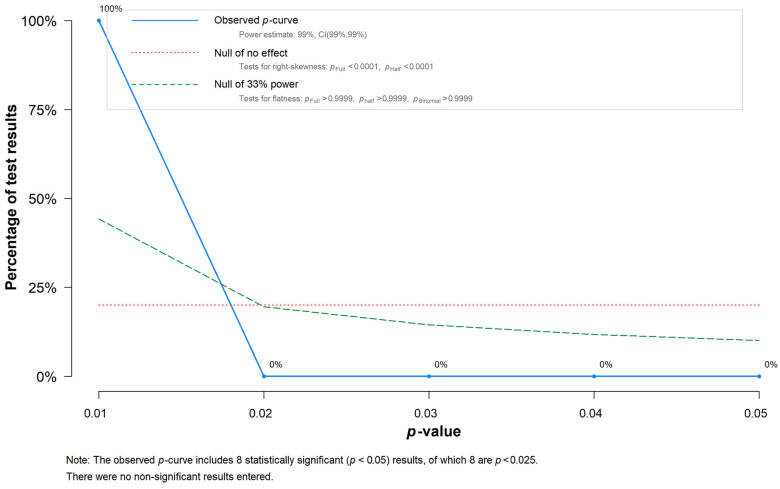
*p*-curve analysis of all studies included in the present meta-analysis.

*p*-curve analysis was conducted on eight significant effect sizes (all studies had *p* ≤ 0.01) and revealed right-skew (*p* < 0.001), which may be suggestive of evidential value among the included findings (Figure 13). However, given the small number of studies, this analysis should be interpreted cautiously and should not be taken as definitive evidence against selective reporting, publication bias, or other reporting-related biases.

## 4. Discussion

The present systematic review and meta-analysis presents an up-to-date, comprehensive evidence synthesis of the existing data on the association between circulating CTRP1 concentrations and T2DM in adults. Across eight eligible studies comprising 525 T2DM cases and 612 non-T2DM controls, circulating CTRP1 concentrations were significantly higher in individuals with T2DM (MD: 185.31 ng/mL; 95% CI: 140.02 to 230.60; *p* < 0.001). Meta-regression analyses indicated that this association was partially influenced by the proportion of male participants in a study and the BMI of the sample. Specifically, higher proportions of male participants and greater mean BMI were associated with significantly smaller differences in CTRP1 concentrations between T2DM cases and controls, suggesting that sex representation and adiposity may partially explain variability in MDs across studies, although they do not fully account for the observed elevation in CTRP1 among individuals with T2DM.

The pleiotropic metabolic effects of CTRP1 link this relatively novel adipokine to the pathogenesis of T2DM, although the exact mechanisms are unclear. Animal studies have shown that overexpression or injection of exogenous CTRP1 into animal models results in improved insulin sensitivity and glycaemia, whilst it can also prevent obesity and hepatic steatosis with reduced calorie intake, increased leptin levels, and increased glucose and fatty acid utilisation [21,22,23,24]. Conversely, CTRP1-knockout mice have been shown to exhibit increased insulin resistance independent of adiposity, higher rates of hepatic gluconeogenesis, higher fasting insulin levels, and lower glucose utilisation [25]. The insulin-sensitising effects of CTRP1 are shown to be mediated via the AMP-activated protein kinase (AMPK) signalling pathway [24] and through reduced serine-1101 phosphorylation in IRS-1 [22]. Given such protective metabolic effects of CTRP1, it has been hypothesised that lower circulating CTRP1 concentrations could be associated with T2DM. However, the present findings indicate significantly higher circulating CTRP1 concentrations in adults with T2DM compared to non-T2DM controls, suggesting either resistance to the protective metabolic effects of CTRP1 or a potential compensatory mechanism in response to the development of T2DM.

Previous human studies have demonstrated a significant, positive correlation between circulating CTRP1 and the homeostasis model assessment of insulin resistance (HOMA-IR) index, indicating increased circulating CTRP1 in insulin-resistant states [50,52,53,55,59]. Indeed, the studies by Xin et al. [55] and Pan et al. [53] demonstrated this positive correlation in adults with T2DM. Their findings suggest that additional CTRP1 release may occur beyond a certain degree of insulin resistance, acting as an insulin-sensitising compensatory mechanism in the diabetic state; however, further studies are required to clarify this association. Of note, Han et al. [51] showed elevated circulating CTRP1 concentrations in adults with pre-diabetes compared to healthy controls, suggesting that the insulin resistance threshold for increased CTRP1 secretion may start at the pre-diabetic stage. Moreover, Kim et al. [60] have demonstrated increased CTRP1 expression in rats injected with tumour necrosis factor-α or interleukin-1β, suggesting that it is possible that the pro-inflammatory state that typically characterises T2DM [61] may contribute to increased CTRP1 expression and secretion. Future research should explore the mechanisms behind compensatory CTRP1 release in the insulin-resistant state and confirm whether CTRP1 resistance additionally contributes to increased circulating CTRP1 concentrations in adults with T2DM. In addition to mechanistic studies exploring the underlying mechanisms, prospective clinical studies should examine the temporal direction of the association between circulating CTRP1 and T2DM in order to elucidate their causal association.

The meta-regression analysis revealed that the mean BMI of the study sample was significantly and inversely associated with the MD in CTRP1 concentrations between the T2DM and non-T2DM groups. Simply put, study samples with higher average BMI tended to report smaller differences in circulating CTRP1 between T2DM and non-T2DM groups. This finding can be partially explained by the increase in white adipose tissue mass in those with higher BMI, which can account for higher expression and secretion of CTRP1 by the accumulated excess white adipose tissue [18,19]. As such, it is possible that the control participants without T2DM, but with a higher BMI, could have increased circulating CTRP1 concentrations; hence, reducing the MD with the circulating CTRP1 concentrations of participants with T2DM. This plausible explanation is consistent with studies showing an independent positive correlation between BMI and circulating CTRP1 concentrations [16,51,54,55] and higher CTRP1 concentrations in participants with obesity [62]. Given the evidence on higher circulating CTRP1 concentrations in adults with T2DM, further research is required to characterise any dysregulation and potential resistance to the protective metabolic effects of CTRP1 in T2DM. For example, the regulation of CTRP1 and insulin receptor substrate 1 (IRS-1) should be further studied in obesity and T2DM since CTRP1 is shown to improve insulin sensitivity by decreasing the phosphorylation of serine-1101 in IRS-1, suggesting that CTRP1 may be secreted as a feedback adipokine to counteract insulin resistance [22].

In addition, the present meta-regression results indicated that studies with a greater proportion of male participants had a lower difference in the circulating CTRP1 concentrations between T2DM cases and non-T2DM controls, although this was still significantly higher in those with T2DM. This finding may be, at least partly, related to the well-established sex-related differences in body fat distribution [63,64,65]. Influenced by sex hormones, women tend to accumulate more subcutaneous adipose tissue in the gluteofemoral region and mammary glands (gynoid body fat distribution), whilst men tend to store excess adipose tissue more in the visceral and subcutaneous abdominal adipose tissue depots (android body fat distribution) [63,64,66]. Of note, surgical removal of visceral adipose tissue has been shown to improve insulin resistance in rats [67] and in patients with severe obesity [68]. Overall, visceral adipose tissue exhibits a stronger link to metabolic dysfunction and insulin resistance compared to subcutaneous adipose tissue due to several proposed mechanisms [1,69]. For example, compared to subcutaneous adipose tissue, visceral fat is associated with greater insulin degradation, greater susceptibility to hypoxia, increased lipolysis and free fatty acid release, as well as a more deleterious adipokine secretion profile [70], which contributes to dyslipidaemia, hepatic insulin resistance, and T2DM [63,64,71,72,73]. In this context, the expression and secretion of CTRP1 by the different adipose tissue depots, particularly in relation to the body fat distribution in adults, merit further research. Interestingly, it has been shown that CTRP1 levels were significantly higher in the plasma and epicardial adipose tissue (a type of ectopic/visceral fat sharing origins with mesenteric and omental adipocytes [74]) of adults with congestive heart failure compared to controls without [75]. Furthermore, the study by Majidi et al. [52] in men with T2DM and without T2DM showed a positive correlation between circulating CTRP1 concentrations and visceral adipose tissue thickness, which tended to be significant.

In addition to BMI and sex, there are several other possible factors, such as ethnicity, duration of T2DM and medication use, which could be contributing to the considerable heterogeneity observed in our meta-analysis. Currently, there is limited evidence on the relationship between circulating CTRP1 and such factors. However, research on this could be guided by relevant research that has explored these factors in relation to other adipokines, particularly the closely related adiponectin. For example, regarding ethnicity, data from adults who participated in the Study of Health Assessment and Risk in Ethnic Groups (a cross-sectional prevalence study of cardiovascular disease risk factors in Canada) showed that circulating adiponectin concentrations were significantly higher in Canadians of European and Aboriginal origin compared to those of South Asian and Chinese origin [76]. Furthermore, the findings of a prospective study from Sweden on newly diagnosed patients with T2DM showed that circulating adiponectin concentrations increased over the 3-year follow-up period, suggesting a temporal relationship between circulating adiponectin and T2DM [77]. Moreover, existing data indicate that circulating adiponectin concentrations can be increased by medications such as statins, angiotensin-converting enzyme inhibitors, angiotensin receptor blockers, thiazolidinediones and glucagon-like peptide-1 agonists [78,79], whilst metformin has been shown to significantly reduce leptin and resistin levels [80]. Other medications such as steroids, beta-blockers, tricyclic antidepressants and antipsychotics have been implicated in the development of drug-induced diabetes and may further dysregulate circulating adipokine profiles [81]. As it is plausible that similar relationships may exist between circulating CTRP1 and factors such as ethnicity, duration of T2DM and medications, targeted research is also required to explore these potential associations.

### Study Limitations

The present systematic review and meta-analysis has certain limitations. Firstly, the number of existing eligible studies, although allowing for a meta-analysis, is relatively small and thus limiting, particularly regarding feasible subgroup analyses. For example, four of the eligible studies included in the meta-analysis were from China, and the other four were from three other countries, a global distribution that is not representative of the worldwide population. As such, the “rest of the world” subgroup contained heterogeneous populations from three different countries, and therefore this analysis should be interpreted with caution. Additionally, the “not reported” subgroup for the T2DM diagnostic method consisted of just one study and its influence should therefore be interpreted cautiously. Although all included studies favoured higher circulating CTRP1 concentrations in T2DM cases, the wide prediction interval indicates substantial between-study heterogeneity and suggests that future studies may observe a broad range of effect sizes. Therefore, the pooled estimate should be interpreted as an average effect across heterogeneous study populations, rather than as a precise estimate expected to apply equally across all settings and populations. Indeed, considerable heterogeneity was identified between the included studies, which remained despite the parameters explored by the performed subgroup analysis and meta-regression. The study by Yagmur et al. [56] was conducted in critically ill patients admitted to the intensive care unit (ICU), a population clinically distinct from the other T2DM cohorts. Circulating CTRP1 concentrations in this setting may reflect acute illness, sepsis, systemic inflammation, or ICU-related treatments, in addition to T2DM status. This may have contributed to clinical heterogeneity and limits direct comparability with non-ICU T2DM populations. However, the leave-one-out sensitivity analysis indicated that the primary finding of higher circulating CTRP1 concentrations in T2DM was not dependent on the inclusion of this study. Furthermore, included studies did not fully account for potential confounders, such as ethnicity, duration of T2DM, glycaemic control, inflammatory states, medication use, and comorbidities (e.g., NAFLD, renal dysfunction/disease and cardiovascular disease). Moreover, the existing eligible studies were cross-sectional and, thus, causality could not be explored. In addition, certain studies reported data in graphical formats or as median (IQR) values, requiring plot digitisation or transformation to mean ± SD values, respectively, for inclusion in the present meta-analysis. Furthermore, the sunset plot indicated high statistical power among the included studies; however, assessment of small-study effects and publication bias remains limited by the small number of studies included in the meta-analysis. Funnel plot asymmetry, Egger’s test, and trim-and-fill methods are known to be underpowered and potentially unstable when fewer than 10 studies are available; thus, these findings should be interpreted with caution. Given the small number of included eligible studies, the meta-regression findings should also be interpreted cautiously and considered exploratory. Finally, the present systematic review included studies that have been published only in the English language, and this may have resulted in the omission of relevant studies published in other languages.

## 5. Conclusions

The present systematic review and meta-analysis offers an up-to-date and comprehensive evidence synthesis, which shows that circulating CTRP1 concentrations are significantly higher in adults with T2DM compared to controls without T2DM. Given its protective metabolic effects, these findings highlight the need for further research focusing on CTRP1 in relation to insulin resistance and T2DM in order to increase the corresponding body of evidence and elucidate the nature of this association. Indeed, prospective clinical studies are needed to explore the temporal direction of the association between circulating CTRP1 concentrations and T2DM. The potential impact on this association of parameters, such as sex, body fat distribution, ethnicity and medication use, should also be explored. Moreover, basic research studies are also required to further elucidate the underlying pathophysiological mechanisms contributing to increased circulating CTRP1 concentrations in T2DM (e.g., underlying pathophysiological mechanisms resulting in a compensatory response to insulin resistance or in resistance to CTRP1). The findings of these clinical and mechanistic studies will determine whether circulating CTRP1 can be used in the future as a potential additional diagnostic or prognostic biomarker in T2DM.

## Figures and Tables

**Table 1:** Search Strategy for Medline (via Ovid) database.

#	Query
1	(“Complement C1q/TNF-related Protein-1” or “C1q/TNF-related protein-1” or “1q (C1q)/tumor necrosis factor” or “CTRP-1” or “CTRP 1” or CTRP1).mp.
2	exp Diabetes Mellitus, Type 2/
3	((typ? 2 or typ? II or typ?2 or typ?II) adj3 diabet*).tw.
4	“diabetes mellitus”.mp.
5	(non insulin* depend* or noninsulin* depend* or noninsulin?depend* or non insulin?depend*).tw.
6	(T2D or T2DM or NIDDM).tw.
7	or/2–6
8	1 and 7

**Table 2:** Key pertinent characteristics of the eight eligible studies included in the present systematic review and meta-analysis.

Authors (Year), [Ref.]	Study Characteristics	Study Group Characteristics		Key Findings
Bai et al. (2017) [50]	Country: China Design: Cross-sectional study CTRP1 Assay: ELISA (BioVendor, Inc., Czech Republic) T2DM diagnostic criteria: WHO (1999) Primary comparison(s): newly diagnosed adults with T2DM vs. age- and BMI-matched participants without T2DM	T2DM group *N* = 124 Age: median 51.0 years (IQR: 43.0–59.0) Sex: 53.2% male BMI: median 25.8 kg/m^2^ (IQR: 22.9–28.4)	Non-T2DM group *N* = 138 Age: median 49.0 years (IQR: 41.0–56.0) Sex: 53.6% male BMI: median 25.0 kg/m^2^ (IQR: 23.0–27.2)	Circulating CTRP1 concentrations were significantly higher in the T2DM group (median: 543.3 [IQR: 430.8–708.8] ng/mL) compared to the control group (308.7 [IQR: 255.2–354.9] ng/mL); *p* < 0.01.
Han et al. (2016) [51]	Country: Republic of Korea (South Korea) Design: Cross-sectional study CTRP1 Assay: ELISA (BioVendor, Inc., Czech Republic) T2DM diagnostic criteria: Fasting blood glucose levels > 126 mg/dL or HbA1c > 6.5% Primary comparison(s): adults with T2DM vs. non-T2DM (not age- or BMI-matched)	T2DM group *N* = 58 Age: not reported Sex: not reported BMI: 25.3 ± 3.1 kg/m^2^ (mean ± SD)	Non-T2DM group *N* = 68 Age: not reported Sex: not reported BMI: 22.45 ± 2.7 kg/m^2^	Circulating CTRP1 concentrations were significantly higher in the T2DM group (mean ± SD: 355.3 ± 341.3 ng/mL) compared to control group (103.7 ± 53.4 ng/mL); *p* < 0.001. Higher circulating CTRP1 concentrations were associated with age, BMI, fasting glucose and FGF21 concentrations. Circulating CTRP1 concentrations were independently and positively associated with fasting blood glucose after adjustment for potential confounders, including BMI, age, sex and medication use.
Majidi et al. (2021) [52]	Country: Iran Design: Cross-sectional study CTRP1 Assay: ELISA (BioVendor, Inc., Czech Republic) T2DM diagnostic criteria: ADA (1997) Primary comparison(s): adult men with T2DM vs. age- and BMI-matched participants without T2DM	T2DM group *N* = 42 Age: median 55 years (IQR: 46.5–59.25) Sex: 100% male BMI: 28.78 ± 4.33 kg/m^2^ (mean ± SD)	Non-T2DM group *N* = 42 Age: median 51 years (IQR: 48–57.25) Sex: 100% male BMI: 27.13 ± 3.72 kg/m^2^	Circulating CTRP1 concentrations were significantly higher in the T2DM group (median: 414.8 [IQR: 368.1–534.9] ng/mL compared to control group (343.9 [IQR: 272.6–392.3] ng/mL; *p* < 0.0001. (Note: CTRP1 values were extracted from Figure 1b of the corresponding published paper using a plot digitiser).
Pan et al. (2014) [53]	Country: China Design: Cross-sectional study CTRP1 Assay: ELISA (BioVendor, Inc., Czech Republic) T2DM diagnostic criteria: ADA (1997) Primary comparison(s): adults with T2DM vs. age- and sex- matched participants without T2DM	T2DM Group *N* = 96 Age: 46.13 ± 2.42 years (mean ± SEM) Sex: 49% male BMI: 24.51 ± 0.90 kg/m^2^ (mean ± SEM)	Non-T2DM Group *N* = 85 Age: 45.25 ± 1.94 years Sex: 51% male BMI: 21.76 ± 0.42 kg/m^2^	Circulating CTRP1 concentrations were significantly higher in the T2DM group compared to control group in both men (mean ± SEM: 378.3 ± 43.93 versus 135.6 ± 15.42 ng/mL, respectively; *p* < 0.001) and women (304.4 ± 30.40 versus 149.8 ± 12.71 ng/mL, respectively; *p* < 0.001).
Shabani et al. (2015) [54]	Country: Iran Design: Cross-sectional study CTRP1 Assay: ELISA (BioVendor, Inc., Czech Republic) T2DM diagnostic criteria: ADA (1997) Primary comparison(s): adult men with T2DM vs. NAFLD vs. participants with both T2DM and NAFLD vs. healthy participants without T2DM nor NAFLD; all groups were matched for age	T2DM only group *N* = 22 Age: 55.95 ± 1.73 years (mean ± SEM) Sex: 100% male BMI: 27.28 ± 0.91 kg/m^2^ (mean ± SEM) T2DM plus NAFLD group *N* = 22 Age: 52.87 ± 1.70 years Sex: 100% male BMI: 30.61 ± 0.84 kg/m^2^	Healthy control group *N* = 21 Age: 53.95 ± 1.62 years Sex: 100% male BMI: 24.76 ± 0.80 kg/m^2^ NAFLD only group *N* = 22 Age: 52.27 ± 1.38 years Sex: 100% male BMI: 29.18 ± 0.50 kg/m^2^	Circulating CTRP1 concentrations were significantly higher in the T2DM (mean: 441.6 [95% CI: 432.5–450.0] ng/mL), NAFLD (377.0 [95% CI: 368.3–386.5] ng/mL) and T2DM plus NAFLD (463.0 [95% CI: 453.2–472.7] ng/mL) groups compared to the healthy control group (313.0 [95% CI: 307.1–318.2] ng/mL; *p* < 0.0001). (Note: CTRP1 values were extracted from Figure 1a of the corresponding published paper using a plot digitiser). Circulating CTRP1 had significant positive correlations with fasting blood glucose and BMI.
Xin et al. (2014) [55]	Country: China Design: Cross-sectional study CTRP1 Assay: ELISA (BioVendor, Inc., Czech Republic) T2DM diagnostic criteria: WHO (1999)	T2DM group *N* = 62 Age: mean 61 years (range 37–79) Sex: 51.6% male BMI: 26.0 ± 2.3 kg/m^2^ (mean ± SD)	Non-T2DM group *N* = 73 Age: mean 58 years (range 31–80) Sex: 47.9% male BMI: 24.0 ± 1.8 kg/m^2^	Circulating CTRP1 concentrations were significantly higher in the T2DM group (mean ± SD: 646.3 ± 154.4 ng/mL) compared to the control group (442.6 ± 165.4 ng/mL); *p* < 0.001.
Xin et al. (2017) [22]	Country: China Design: Cross-sectional study CTRP1 Assay: ELISA (BioVendor, Inc., Czech Republic) T2DM diagnostic criteria: WHO (1999) Primary comparison(s): adults with T2DM vs. age- and sex-matched participants without T2DM; participants scheduled for elective abdominal operations	T2DM group *N* = 35 Age: median 54 years (range 31–63) Sex: 65.7% male BMI: 29.3 ± 2.7 kg/m^2^ (mean ± SD)	Non-T2DM group *N* = 35 Age: median 51 years (range 26–67) Sex: 60.0% male BMI: 24.7 ± 1.5 kg/m^2^	Circulating CTRP1 concentrations were significantly higher in the T2DM group (mean ± SD: 712.3 ± 98.7 ng/mL) compared to the control group (548.5 ± 121.9 ng/mL); *p* = 0.030. Circulating CTRP1 concentrations were negatively correlated with insulin resistance.
Yagmur et al. (2019) [56]	Country: Germany Design: Retrospective cohort study CTRP1 Assay: ELISA (BioVendor, Inc., Czech Republic) T2DM diagnostic criteria: Not reported Primary comparison(s): Critically ill patients vs. healthy controls; sepsis vs. non-sepsis group; high disease severity (APACHE score ≥ 10) vs. lower disease severity (APACHE score < 10); patients with vs. without pre-existing T2DM; participants were critically ill patients treated in a medical ICU setting	T2DM group *N* = 64 Age: not reported Sex: not reported BMI: not reported	Non-T2DM group *N* = 150 Age: not reported Sex: not reported BMI: not reported	Circulating CTRP1 concentrations were significantly higher in critically ill patients (median: 747.1 ng/mL) versus the healthy controls (316.3 ng/mL); *p* < 0.001. Circulating CTRP1 concentrations were significantly higher in patients with sepsis (median: 779.6 ng/mL) versus the patients without sepsis (574.2 ng/mL); *p* = 0.006. Circulating CTRP1 concentrations were significantly higher in ICU patients with pre-existing T2DM (median: 833.5 ng/mL) versus those ICU patients without pre-existing diabetes (626.3 ng/mL); *p* = 0.004.

ADA: American Diabetes Association; APACHE: Acute Physiology and Chronic Health Evaluation; BMI: body mass index; CI: confidence interval; ELISA: enzyme-linked immunosorbent assay; FGF21: Fibroblast Growth Factor 21; HbA1c: glycated haemoglobin; ICU: intensive care unit; IQR: interquartile range; NAFLD: non-alcoholic fatty liver disease; T2DM: type 2 diabetes mellitus; SEM: standard error of the mean; SD: standard deviation; WHO: World Health Organization.

**Table 3:** Summary of findings for primary outcome: Circulating CTRP1 concentrations in T2DM cases versus controls and certainty of the corresponding evidence using the Grading of Recommendations Assessment, Development, and Evaluation (GRADE) approach.

Outcomes (ng/mL)	MD (95% CI)	Number of Participants (Studies)	Certainty of the Evidence (GRADE)	Comments
CTRP1	185.31 (140.02 to 230.60)	1137 (8 trials)	⨁◯◯◯ Very low ^a,b,c^	Circulating CTRP1 concentrations were statistically higher in T2DM cases compared to non-T2DM, but caution should be adopted when interpreting this finding.
Key: CI: confidence interval; CTRP1: C1q tumour necrosis factor-related protein 1; MD: mean difference; T2DM: type 2 diabetes mellitus				
GRADE Working Group grades of evidence High certainty: We are very confident that the true effect lies close to the estimate of the effect. Moderate certainty: We are moderately confident in the effect estimate: the true effect is likely to be close to the estimate of the effect, but there is a possibility that it is substantially different. Low certainty: Our confidence in the effect estimate is limited: the true effect may be substantially different from the estimate of the effect. Very low certainty: We have very little confidence in the effect estimate: the true effect is likely to be substantially different from the estimate of effect.				

**Explanations**: ^a^: Downgraded once: majority of included trials judged to have an overall unclear/high risk of bias and to be of fair/poor quality. ^b^: Downgraded once: considerable degree (85.9%) of between-study heterogeneity. ^c^: Downgraded once: small number of studies, small sample size, and dependence upon observational study designs.

## Data Availability

No new data were created or analysed in this study. Data sharing is not applicable to this article.

## Supplementary Materials

The following supporting information can be downloaded at https://www.mdpi.com/article/10.3390/ijms27156775/s1. Reference [82] is cited in the supplementary materials.
